# Genome profiling of ovarian adenocarcinomas using pangenomic BACs microarray comparative genomic hybridization

**DOI:** 10.1186/1755-8166-1-10

**Published:** 2008-05-20

**Authors:** Donatella Caserta, Moncef Benkhalifa, Marina Baldi, Francesco Fiorentino, Mazin Qumsiyeh, Massimo Moscarini

**Affiliations:** 1 Oby/Gyn Dept. Saint Andrea Hospital, University of Roma La Sapienza, Rome, italy; 2 Genetics & IVF, A TL R&D Laboratory, La Verrière france & UNILABS Laboratories, Geneva, Switzerland; 3 Molecular Biology & Cytogenetics, Genoma Laboratories, Rome, italy; 4 Cytogenetics Laboratory, SiParadigm Laboratories, 690 Kinderkamack Rd, Oradell, NJ, USA

## Abstract

**Background:**

Routine cytogenetic investigations for ovarian cancers are limited by culture failure and poor growth of cancer cells compared to normal cells. Fluorescence *in situ *Hybridization (FISH) application or classical comparative genome hybridization techniques are also have their own limitations in detecting genome imbalance especially for small changes that are not known ahead of time and for which FISH probes could not be thus designed.

**Methods:**

We applied microarray comparative genomic hybridization (A-CGH) using one mega base BAC arrays to investigate chromosomal disorders in ovarian adenocarcinoma in patients with familial history.

**Results:**

Our data on 10 cases of ovarian cancer revealed losses of 6q (4 cases mainly mosaic loss), 9p (4 cases), 10q (3 cases), 21q (3 cases), 22q (4 cases) with association to a monosomy X and gains of 8q and 9q (occurring together in 8 cases) and gain of 12p. There were other abnormalities such as loss of 17p that were noted in two profiles of the studied cases. Total or mosaic segmental gain of 2p, 3q, 4q, 7q and 13q were also observed. Seven of 10 patients were investigated by FISH to control array CGH results. The FISH data showed a concordance between the 2 methods.

**Conclusion:**

The data suggest that A-CGH detects unique and common abnormalities with certain exceptions such as tetraploidy and balanced translocation, which may lead to understanding progression of genetic changes as well as aid in early diagnosis and have an impact on therapy and prognosis.

## Background

A number of strategies have been used for early detection of ovarian cancer and follow-up. CA-125 tumor marker investigation and trans vaginal ultrasound are the most common used procedures [1,2]. An increase of CA-125 marker has been shown to predate clinical or scan evidence of relapse in approximately 70% of patient with ovarian cancer [3]. For Genome disorders investigation classical cytogenetic, fluorescence in situ hybridization and comparative genome hybridization methods were applied in cancers [4-6]. Recent studies suggest that genomic changes can be useful for cancer grading [7]. For example, a study by Simon et al. [8] showed that breakpoints in regions 1p3 and 11p1 are important early events and distinguish a class of tumors associated with poor prognosis in ovarian adenocarcinoma. Genomic changes in ovarian cancers were investigated by using various techniques, each with its own limitations. For example fluorescence *in situ *hybridization was used by Liehr et al. [9], on 25 cases of ovarian carcinomas using alphoid probes noting loss of chromosomes 17 and 20 and gain of chromosomes 7, 1, 8 and 11 [10]. Classical comparative genomic hybridization was also used but is limited in their resolution [11-14]. Gene expression and proteomics arrays were applied to assess gene expression changes which could be due to both genotype changes, regulation pathway changes, or epigenetic [15-19]. Single nucleotide polymorphism arrays were also used recently to detect some micro deletions and amplifications in serous ovarian carcinomas [20,21].

Array comparative genome hybridization (A-CGH) using spotted bacterial artificial chromosomes (BACs), phage artificial chromosomes (PACs), cDNAs or oligonucleotides provides distinct advantages in specificity, sensitivity and high resolution genome analysis [22-28]. Microarray CGH combined to immunohistochemical analysis was applied for genes amplification and prognostic markers analysis in ovarian cancer [29]

Micro arrays provide distinct advantages over conventional and molecular cytogenetics (pre and post natal, cancer and oncology) analysis because they have the potential to detect the majority of microscopic and sub microscopic chromosomes changes from any DNA sources with or without whole genome amplification [30,31]. Other advantage of A-CGH is the increase in resolution that can be achieved compared to chromosome-based CGH. The only limitations of CGH (both conventional and A-CGH) is the inability to detect polyploidy or balanced chromosome abnormalities. But polyploidy can be easily detected by FISH, micro satellite analysis or flow cytométrie and balanced translocations will still be detectable using classic cytogenetics or FISH.

## Results

**Figure 1:**
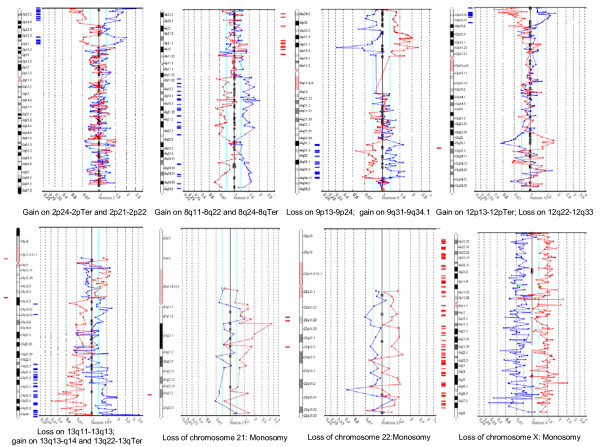
examples of chromosomes profiles and abnormalities.

**Figure 2:**
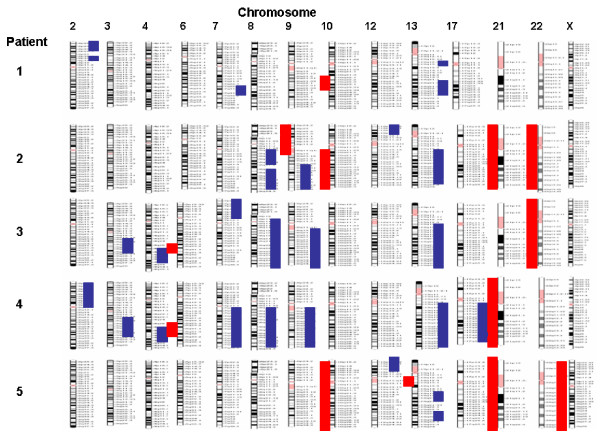
Genome profiling of patients 1 to 5.

**Figure 3:**
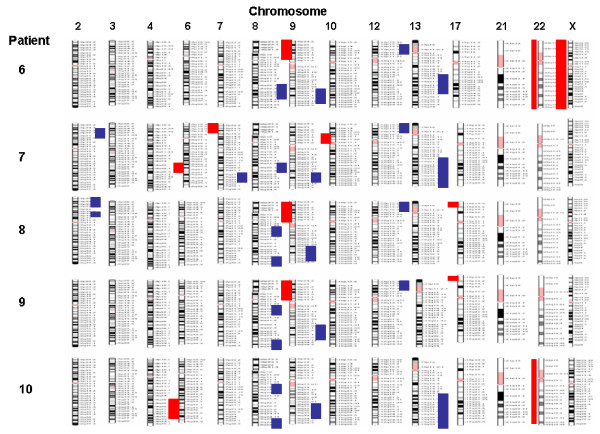
Genome profiling of patients 6 to 10.

**Figure 4:**
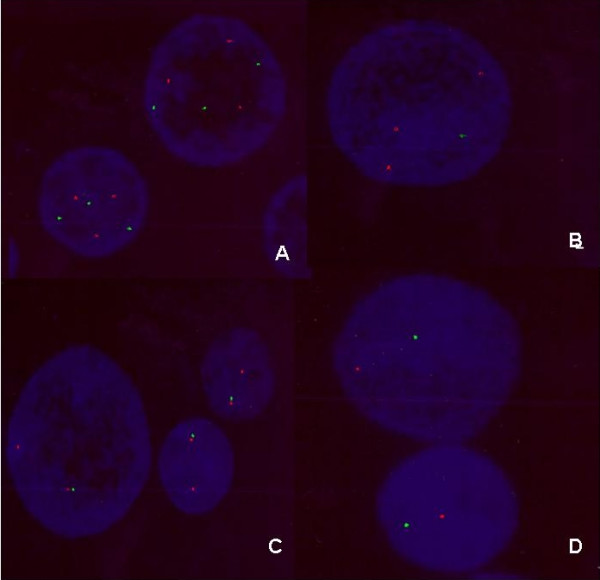
**Examples of FISH data using BACs probes**. A) Case 2: trisomy 8q and 13q B) Case 5: trisomy 12p and monosomy X. C) Case 6: disomy 9q monosomy 9p D) Case 10: monosomy 6q monosomy 22.

The main endocrinological observation in these patients was an excess of sex steroids production. Histological findings revealed 9 of 10 patients with serous papillary cystadenocarcinoma (eight grade III and one grade II), the remaining patient showed endometrial carcinoma with papillary aspects and squamous metaplasia (Table 1). The data we obtained from A-CGH showed that all ten patients have an abnormal genome profile with both unique and common changes (Table 2 and Figures 1, 2, 3). The most common findings were loss of 6q (4 cases with mosaic loss of 6q), 9p (4 cases), 10q (3 cases), 21q (3 cases), 22q (4 cases) and gain of 8q and 9q (occurring together in 8 cases) and gain of 12p. Two cases with monosomy X were observed. A micro deletion of 17p terminal was observed in 2 cases. Some cases showed also a genomic profile with total or mosaic segmental gain on chromosomes 2p, 3q, 4q, 7q and 13q. Seven of 10 patients (1, 2, 4, 5, 6, 7, and 9) were analyzed by FISH for aneuploidy confirmation using a cocktail of bacs probes for each specific abnormal chromosome region. We were not able to analyze the remaining 3 patients because of the limited material. The obtained data by FISH was in concordance with the CGH array except the patient number 1 and 9. For the 2 patients we couldn't confirm the mosaic loss of 18 q for patient 1 and the segmental loss of 12q22-12q23 for patient 9 (see Fig 4).

**Table 1:** Hystological Findings

**N° case**	**Name (initials)**	**Age**	**Grading**	**Hystological Diagnosis**
1	PG	44	pT3c pNO pMX; Stage III C grade G2 (AICC 2002)	cystoadenocarcinoma
2	TS	37	PTla pNO pMx Stage lA (AlCC2002)	Endometrial carcinoma of the right ovary with papillary aspects and squamous metaplasia
3	VM	65	PTI b pNO G3 Stage IB (AlCC 2002)	Cystoadenocarcinoma serous Papillary left and right ovary G 3
4	LR	84	PT2a pNX pMX Stage 2A G3 (AlCC 2002) Stage IIA FIGO)	Cystoadenocarcinoma, serous papillary left and right ovary G3
5	AW	75	Stage IV G3	Cystoadenocarcinoma, serous papillary G3
6	FR	73	pT2a pNI pMX Stage IIIC (AlCC 2002)	Cystoadenocarcinoma serous papillary left ovary G3
7	PL	50	PT2a pNX pMX Stage 2A G3 (AlCC 2002) Stage IIA FIGO)	Cystoadenocarcinoma serous papillary left and right ovary G3
8	MA	64	PT2a pNX pMX Stage 2A G3 (AlCC 2002) Stage IIA FIGO	Cystoadenocarcinoma serous papillary left and right ovary G3
9	MF	68	PT3c pN1 pMX Stage mc G3 (AlCC 2002)	Cystoadenocarcinoma serous papillary left ovary
10	WDT	45	PT3c pN1 pMX Stage IIIC G3 (AlCC 2002)	Cystoadenocarcinoma serous papillary left and right ovary G3

**Table 2:** Genome profile results of the 10 patients

**N°**	**Name**	**Segmental gain**	**Segmental loss**	**Other abnormalities**
l	PG	Segmental gain on 2p, 7q, and 13 q arms	Mosaic segmental loss on 10 q and 18 q arms	
2	TS	Segmental gain on 8q, 9q, 12p, and 13q arms	Total loss of 9p and 10q arms	Monosomy 21 and 22
3	VM	Mosaic segmental gain on 3q and 4q arms Total gain of 7q, 8q, 9q, 13q arms	Mosaic segmental loss on 6q arms	Monosomy 22
4	LR	Mosaic segmental gain on 3q and 4q Total gain of 2p, 7q, 8q, 9q 13q and 17q arms	Mosaic segmental loss on 6q arms	Monosomy 21
5	AW	Segmental gain on 12p arms	Total loss of chromosome 10	Segmental disorder of chromosome 13 monosomy 21 and X
6	FR	Segmental gain on 8q, 9q, 12p and 13q arms	Segmental loss on 9p arm	Monosomy 22 and X
7	PL	Segmental gain on 2p, 7q, 8q, 9q, 12p,13q arms	Mosaic segmental loss on 6q, 7p and 10p arms	
8	MA	Segmental gain on 2p, 8q and 9q arms Mosaic segmental gain on 12p arm	Segmental loss on 9p arm	Cryptic loss of tel 17p
9	MF	Segmental gain on 8q and 9q arms Mosaic segmental gain 12p arm	Segmental loss on 9p arm	Cryptic loss of tel 17p
10	WDT	Segmental gain on 8q, 9q and 13q arms	Segmental mosaic loss on 6q arm	Monosomy 22

## Discussion

While early detection predicts treatment success, fewer than 25% of ovarian cancers are currently detected at stage I [32]. Approaches to ovarian screening include transvaginal sonography, serum CA-125 markers or both, but these include limitations in sensitivity and specificity [33]. A rapid fall of CA 125 during chemotherapy predicts a favorable prognosis and could be used to redistribute patients on randomized clinical trials [34]. Gene expression and proteomic arrays were used to identify markers that can be used in combination with the clinical picture for early detection [35].

We observed both qualitative and quantitative differences as well as similarities between our data from array CGH on these ovarian tumors from familial cases and abnormalities noted by classical G-banding techniques or other molecular cytogenetic methods in the literature [36]. We noted for example 8 of the ten cases with gain of 8q and 9q.

G-band studies remain the gold standard in cytogenetics laboratories, but most such studies were carried on more advanced cancers and reveal significant abnormalities related to acquision of nuclear instability [37,29,41]. Tharapel et al [42] reported a correlation between chromosome abnormalities and cancer stages such as presence of trisomy 7 and 10 in early stages of ovarian adenocarcinoma. In one of the largest studies, Tibiletti et al. [43] performed cytogenetic characterization of a cohort of 114 untreated ovarian epithelial tumors and concluded that there are three groups: "the first group included abnormalities common to all tumor classes (losses of chromosomes 6, 8, 10, 11, 15, 16, 17, 18, 19, 20, 21, 22, and X; gains of chromosomes 1, 3, 5, and 12 and 6q24-6qter deletions); the second group with specific abnormalities present in malignant but not in benign tumors (losses of chromosomes 2, 7, 13, and 14; gains of chromosome 4 and chromosome markers); and the last group included abnormalities unique to invasive carcinomas (loss of chromosome 4; gains of chromosomes 2, 7, 8, 9, 10, 16, 17, 18, 19, 20, and 21; 6q16-6q24 deletions; rearrangements of 3p, 3q, 13q, and 21q regions). The analysis of 17 sporadic primary ovarian carcinomas by a combination of spectral karyotyping, CGH and expression microarrays and when the distribution of aberrations was normalized with respect to relative genomic length, chromosomes 3, 8, 11, 17 and 21 had the highest frequencies [44]. From in vitro murine model for ovarian cancer the application of microarrays CGH revealed genomic imbalances of chromosomes 4, 5, 7, 8, 9, 11, 15, 17 and X [45].

Aneuploidy assessment in interphase cytogenetics of chromosome 1, 11, 17 and X by fluorescence *in situ *hybridization (FISH) of 92 epithelial ovarian tumors identified numerical aberrations in 67% of mucinous carcinoma and 82% of invasive serous carcinomas but without significant relationship with either stromal invasion or tumor type except for monosomy X which was noted in invasive serous carcinoma [46]. An investigation of 7 stage III ovarian serous cancers by comparative genomic hybridization (CGH) revealed chromosomal aberrations in six patients with certain repeated changes including increased copy numbers of 1q, 2p, 2q, 3q, 6q, 8q, and 12p, and loss of 18q and X [47]. CGH analysis of malignant peripheral primitive neuroectodermal ovarian tumors revealed different chromosomal abnormalities including loss of chromosomes 1p, 1q, 4q, 6p, 6q, 7q, 8q, 13q and 19q; as well as gain of 1q, 2p, 7p, 9q, 18q and Xq [48-50]. The use of CGH and tissue microarrays in ovarian carcinoma [51] showed a frequent chromosomal over presentation on 2q, 3q, 5p, 8q, 11q, 12p, 17q and chromosome 20 combined to an amplification of 59 different oncogenes loci.

Our data showed a loss of chromosome 10 or 10q material in three cases and 6q material in 4 cases. Loss of heterozygosity (LOH) on 10q23 involving PTEN tumor suppressor gene is noted in about 30% of adenocarcinoma and was suggested as an important event in the progression of endometriosis to ovarian adenocarcinoma [52]. By FISH analysis Trisomies 1, 7 and monosomies 9 and 17 were observed in endometriosis, ovarian endometrioid adenocarcinoma and normal endometrium but the frequency of aneusomic cells was significantly higher in ovarian endometrioid carcinoma [10]. This may reflect an expansion of aberrant cells clones already present in endometriosis during the progression to cancer. Levan et al. [13] examined 98 endometrioid adenocarcinomas by CGH and reported 39 chromosomal regions displaying frequent DNA copy number alterations. The analysis of 81 ovarian cancers for LOH by Obata and Hoshiai [53] showed 60% with LOH on chromosome 6q and 40% on 10q. Their data supported the hypothesis that endometrioid and ovarian carcinoma may arise though malignant transformation of endometriotic lesions.

Previous cytogenetics studies of adult germ cells tumors (GCTs) showed that 12p abnormalities are common phenomenon in more than 80% of GCTs. Our data showed 6 out 10 patients with segmental gain of 12 and mainly trisomy 12p. The presence of three or mores copies of 12p may predict resistance to chemotherapy and treatment failure [54].

In our data we observed 3 cases with monosomy 21 and 4 cases with monotony 22. The profiling of human chromosome 22 in ovarian carcinoma by Bentkiewicz et al [55] using high resolution gene copy and expression analysis detected 11 of 18 cases (60%) with heterozygous deletions with various sizes in chromosome 22q and one case with total monosomy 22q. One of the major observations in this study is the presence of segmental gain of 8q and 9q in eight of 10 cases. Several of these cases also had 9p loss. To predict a potential markers of chemo resistant disease, the analysis of chromosomal changes in serous ovarian carcinoma by high resolution array CGH [14] revealed a frequent increase in DNA copy number on 1p36, 3q26, 8q24, 10q26, 12p11, 20q13 and 21q22 and frequent loss on 4p12, 5q13, 7q11, 8p23, 14q32, Xq13 and Xq21. From these data Kim et al [14] conclude that the most reliable combination of chromosomal aberrations for detecting chemo resistant disease was the loss on 13q32.1, 8p21.1. and 16p13 [56].

For genome profiling and high resolution molecular karyotyping, array comparative genome hybridisation (array CGH) methods appear to be far better as they do not suffer from dependence on having metaphase preparations and have much higher sensitivity and specificity for subtle genomic changes. Constitutional deletions as small as 40 kb have been detected using an array encompassing a 7 Mb interval of chromosome 22 with 90% coverage [57]. A-CGH can also provide a technically less demanding and more sensitive assay than classic CGH or even routine cytogenetics. A-CGH use is limited by the cost effective of this technology in routine cytogenetics laboratory and also by the ability to detect certain abnormalities such as tetraploidy and balanced translocations

## Conclusion

It appears likely that in the next few years, array based CGH will become routinely used in clinical cytogenetics. The profiling studies of ovarian cancer by molecular karyotyping and Multiplex ligation-dependent probe amplification [58], expression micro arrays and MicroRNA signature [16,19] and proteome analysis will open the way to more exhaustive and systematic representation of the disease and will provide valuable information that may be helpful to elucidate the mechanism and the evolution of ovarian cancer [17].

## Materials and methods

Tumor tissues were available from ten women with a familial history of ovarian carcinoma who underwent surgery for ovarian masses which required hysterectomy with bilateral ovariectomy at the University of Rome La Sapienza, Saint Andrea Hospital. The mean age of the ten patients studied was 60.5 yrs old.

We used human genomic micro arrays containing 2700 human/BAC/PAC clones (Human BAC Array-System, Perkin Elmer. USA). This array includes subtelomeric regions as well as critical areas spaced roughly 1 Mb along each of the human autosomes as well as the X and Y chromosomes. For consistency and increased sensitivity and specificity, the arrayed clones were printed in duplicate. 2 μg each of the test DNA extracted from the tumor samples and control DNA (female from Promega, Madison, WI) were digested with 80 units of EcoR1 at 37°C overnight and then purified by Zemo Research's column (Orange, CA, USA). The test and reference DNAs were labeled with Cy3 and Cy5 using random prime labeling kit (Invitrogen, Carlsbad, California) to obtain a labeled probe size averaging 100 to 500 bp in length. Cy5 labeled test DNA and Cy3 labeled reference DNA were co-precipated with 65 μg of Cot-1 DNA and 35 μg of Salmon sperm DNA. Forward and reverse hybridization switching of dyes was performed to address issues related to dye specificity and strength. Then the probes were dissolved in 10 μl of distilled water and mixed with 50 μl of hybridization solution (50% formamide, 10% dextran sulphate in 2× SSC). The hybridization mix then was denatured at 73°C for 12 min and followed by 40 min al 37° for annealing. For hybridization and washing we used an automated system (Tray Mix) based on chaotic hybridization approach and developed by BIOTRAY sarl (69007 Lyon France).

Hybridized and washed array slides were analyzed with InnoScan 700A scanner (INNOPSYS, 313901 Carbonne, France). The software recognizes the regions of fluorescent signal, determines signal intensity and compiles the data into a spreadsheet that links the fluorescent signal of every clone on the array to the clone name, its duplicate position on the array and its position in the genome. The software was also used to normalize the Cy5:Cy3 intensity ratios for each data point. Normalization was such that the summed Cy5 signal equals the summed Cy3 signal. The normalized Cy3:Cy5 intensity ratios were computed for each two slides and plotted together for each chromosome. The linear order of the clones in reconstituted in the ratio plots consistent with an ideogram, such that the p terminus in to the left and the q terminus in to the right of the plot.

For data analysis we assign a ratio plot such that gains in DNA copy number at particular locus are observed as the simultaneous deviation of the ratio plots from a modal value of 1.0, with the blue ratio plot showing a positive deviation (upward) while the red ratio plot shows a negative deviation at the same locus (downward). DNA copy number losses show the opposite pattern. In selected cases, fluorescent in situ hybridization (FISH) was used to confirm the A-GCH findings.

## Competing interests

The authors declare that they have no competing interests.

## Authors' contributions

All authors read and approved the final manuscript
